# Effect of tin oxide particle size on epoxy resin to form new composites against gamma radiation

**DOI:** 10.1038/s41598-024-78608-8

**Published:** 2024-11-13

**Authors:** Mohamed Elsafi, Esraa H. Abdel-Gawad, Mohamed A. El-Nahal, M. I. Sayyed

**Affiliations:** 1 Physics Department, Faculty of Science, Alexandria University, Alexandria, Egypt; 2 Environmental Studies Department, Institute of Graduate Studies and Research, Alexandria University, Alexandria, Egypt; 3 Department of Physics, Faculty of Science, Isra University, Amman, Jordan; 4 Renewable Energy and Environmental Technology Center, University of Tabuk, Tabuk, 47913 Saudi Arabia

**Keywords:** Radiation shielding, Epoxy resin, SnO nanoparticles, Lead-free shielding, Physics, Nuclear physics, Experimental nuclear physics

## Abstract

The aim of the present study is to assess the shielding performance of a novel lead-free epoxide material against ionizing radiation. The effect of variation in particle size and concentration of tin oxide (SnO), which was added to epoxy resin polymer (ER), on its radiation shielding properties has been investigated in this research. Ten samples of ER samples incorporated with different concentrations (0%,20%,40%,60%) of SnO microparticles, nanoparticles, and both sizes combined were prepared and assessed. The linear attenuation coefficients (LAC) were measured experimentally through the collimated gamma-ray beam at 0.0595 MeV, 0.6617 MeV, 1.1730 MeV, and 1.330 MeV emitted from Am-241, Cs-137 and Co-60, respectively (to cover all energy range of gamma rays) for all samples with various concentrations and particle sizes of SnO. The other radiological shielding parameters such as half value layer (HVL), tenth value layer (TVL), and radiation protection efficiency (RPE) were estimated and compared for all different samples. The results prove that the increasing of the concentration and reducing the particle size of SnO leads to the enhancement of the radiation protection properties of the ER polymer. Moreover, it was observed that the incorporation of SnO micro- and nanoparticles together improves the radiation shielding properties of ER samples. Conclusively, the reinforcing of ER polymer material matrix by micro/nanoparticles of SnO as composite with enhanced radiation shielding specifications was highlighted.

## Introduction

The ionizing radiation effect has become of excessive worry owing to its deterministic and stochastic impacts. Effective shielding technologies in wearing/storage are now necessary to protect radiation workers from the ever-increasing levels of ionizing radiation in several fields such as generating nuclear power, mining, food production, industrial applications, and medical procedures. Workers may be exposed to radiation when performing various tasks, including handling, preparation, administration, transportation, etc. Ongoing research is moving in the direction of developing suitable materials for radiation shielding in order to meet the mandatory requirements for reducing the harmful effects of ionizing radiation, primarily for the persons operating inside radiation-prone locations^1,2^.

It is well known that high atomic number and density are necessary for effective shielding against gamma radiation in order to provide effective radiation attenuation. Over decades, lead (Pb) and compounds including lead have been exploited widely in radiation protection, despite that, the toxicity of these materials to persons and the environment is a major worry, necessitating the use of hygiene precautions and proper disposal of waste after use. Additionally, lead is expensive and inconvenient to wear for extended periods^3^.

Contrary to the past, composites have begun to grow as shielding materials. Many researchers have focused on merging nanoparticles with numerous materials to produce different types of composites with improved properties. In those complex compounds, nanoparticles (as a filler) increase the likelihood that radiation will interact with matter and diffuse more uniformly and consistently throughout the host material matrix (e.g., polymers, cement, ceramics, alloys, or any other inexpensive materials). This is because of the large interaction surfaces and small particle sizes of the nanoparticles. The produced composites in such cases are considered excellent shielding materials. This is because they have better mechanical, physical, and radiation-shielding qualities. In addition, they are affordable, durable, practical, and lighter^4–7^.

Polymers, in particular, with suitable fillers have been proposed as competent alternatives over any other radiation shielding material because polymers exhibit superior electrical insulation capabilities, and excellent durability thanks to their high heat resistance and chemical stability, besides their reasonable cost and lightness^8^. One of the polymers that is frequently utilized for non-Pb shielding materials is epoxy resin because of its widespread availability, simplicity in preparation, affordability, and resistance to high-intensity radiation damage^9^. Despite its merits, epoxy resin can only block some of the radiation because they are not very dense. To get over this drawback, epoxy resin can be utilized in radiation shielding by incorporating it with dense substances like metal oxides which ultimately leads to greater attenuation effectiveness. In this combination, epoxy resin acts as a matrix to create composites including nanoparticles of a metal oxide to augment its shielding performance^10–15^.

One of the catching attention metal oxides is tin oxide (SnO). It possesses a number of desirable features, including superior mechanical, chemical, and thermal stabilities^16^. Its features proposed it successfully for a widespread zone of applications such as solar cells^17^, gas sensing^18–20^, photocatalysis^21^, and infrared wearable fabric^22^. A continuation of those applications, the current study aims to utilize SnO in the area of radiation shielding as it has a high atomic number and density of $\:6.45\:g/cm$, besides, SnO is easily produced in many shapes (e.g., bulk, nano-sized, or micro-sized materials)^23^ offering versatility in exploiting it in the field of radiation shielding. This study investigates SnO’s performance as a protective supplementary material in improving the shielding capabilities of epoxy resin. So far, no earlier research have investigated the effect of SnO particle size on the radiation attenuation capability of epoxy resin for radiation shielding applications.

Considering the current work, ten samples of epoxy resin (as a matrix) with micro-sized, nano-sized as well as the two sizes combined of SnO (as a filler) were prepared and the radiation shielding performance of the samples was reported by determined the attenuation parameters experimentally using HPGe-detector and different gamma-ray souces emits energies from 0.0595 to 1.333 MeV.

## Materials and methods

### Preparation

**Fig. 1:**
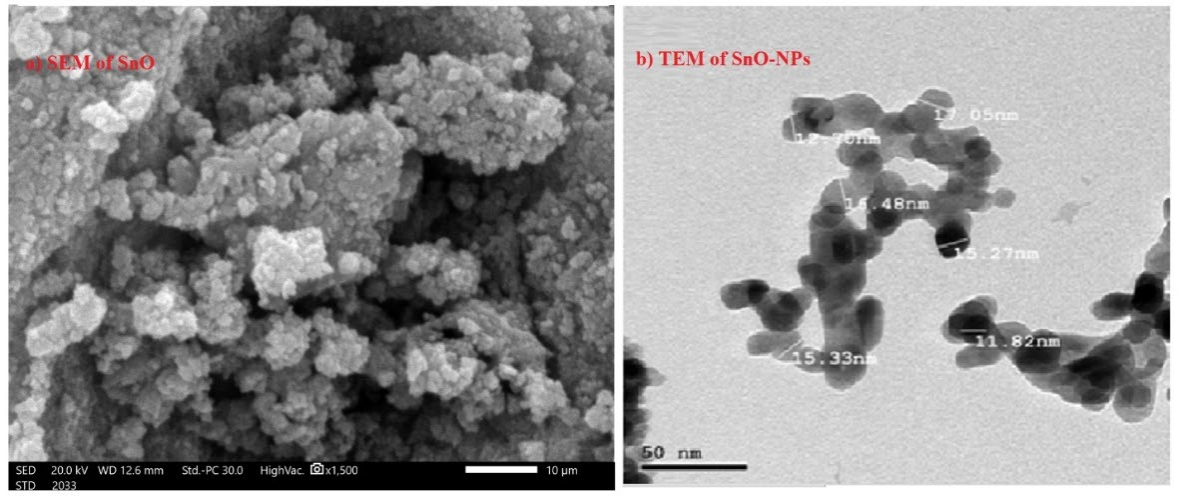
The morphology of SnO-micro and nanoparticles, (**a**) SEM image of micro-SnO (**b**) TEM image of nano-SnO.

Epoxy resin (ER) was used as a matrix composite, while micro- and nanoparticles of tin oxide (SnO) were used as filler in the composite. ER was purchased from a store and it is a product of Turkish origin called erco epoxy resin. It is a transparent liquid accompanied by a hardener that is added at a rate of $$\:50\%$$ of the amount of epoxy resin added (or at a ratio of 2:1). The nature of epoxy resin is DGEBA (bisphenol A) type. For tin oxide, the SnO particles were supplied from Nano-Gate company in Egypt, the shape and size of the SnO microparticles and nanoparticles used in this work were determined by scanning electron microscope (SEM) as shown in Fig. 1, where it was found that the average size of the SnO microparticles was $$\:30\pm\:5$$$$\:\mu\:m$$ and the average size of the SnO nanoparticles was $$\:20\pm\:5$$$$\:nm$$, while the shape of the particles in both sizes was spherical.

The ER composites were fabricated according to the mass percentages, shown in Table 1, where the ER and their hardener were added with micro and nano SnO in a container and stirred well until the produced composite became homogeneous. Then the composites were poured into Polytetrafluoroethylene (PTFE) plastic molds with 2 cm diameter and $\:2\:cm$ thickness and left to dry. After it dried, it was wiped and its surface is made equal to obtain a constant thickness of the composite and some thermal and mechanical properties were studied.

**Table 1:** The mass fraction and codes of the prepared polyester composites.

Sample code	Particle size	Weight fraction (wt., %)			Density, g.cm^− 3^
		Epoxy resin	SnO		
			Micro-particles	Nano-particles	
ER-SnO$\:0\%$		$\:100$	$\:0$	$\:0$	1.191
ER-SnO$\:20\%$	Micro	$\:80$	$\:20$	$\:0$	1.432
	Nano		$\:0$	$\:20$	1.439
	$\:50\:\text{M}\text{i}\text{c}\text{r}\text{o}\:+\:50\:\text{N}\text{a}\text{n}\text{o}$		$\:10$	$\:10$	1.445
ER-SnO$\:40\%$	Micro	$\:60$	$\:40$	$\:0$	1.78
	Nano		$\:0$	$\:40$	1.786
	$\:50\:\text{M}\text{i}\text{c}\text{r}\text{o}\:+\:50\:\text{N}\text{a}\text{n}\text{o}$		$\:20$	$\:20$	1.791
ER-SnO$\:60\%$	Micro	$\:40$	$\:60$	$\:0$	2.346
	Nano		$\:0$	$\:60$	2.352
	$\:50\:\text{M}\text{i}\text{c}\text{r}\text{o}\:+\:50\:\text{N}\text{a}\text{n}\text{o}$		$\:30$	$\:30$	2.358

### Radiation shielding measurements

**Fig. 2:**
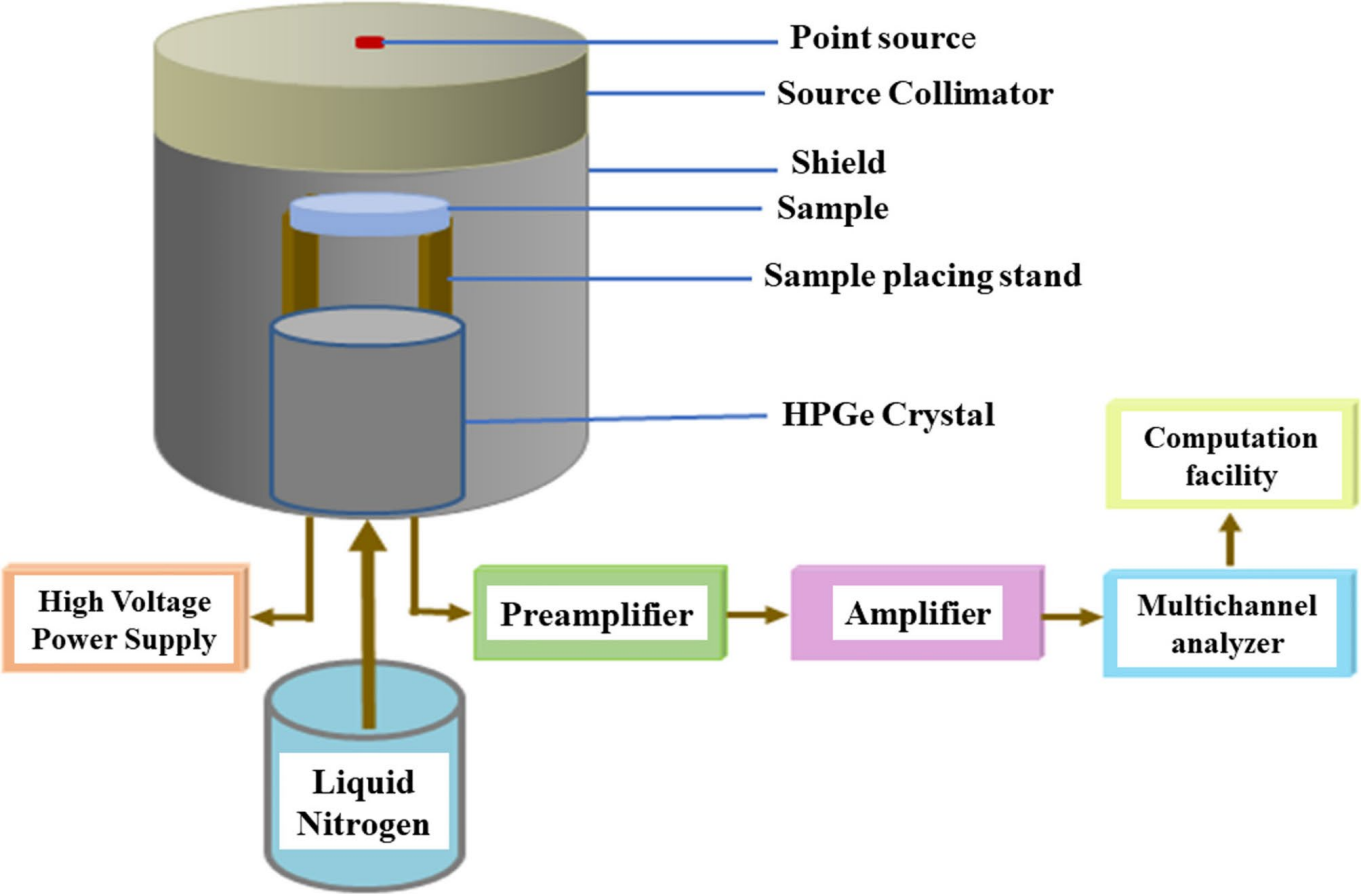
Experimental Setup of measuring the coefficient of attenuation.

The attenuation measurements of the prepared epoxy resin (ER) samples were determined experimentally using HPGe detector and different point sources including Cs-137 ($\:0.06617\:MeV$), Co-60 ($\:\:1.173$ and $\:1.333\:MeV$), and Am-241 ($\:0.0595\:MeV$). The relative efficiency of the detector was 24% as well as the energy resolution was 1.96 keV at 1.333 MeV. The mechanism of measuring is illustrated in Fig. 2 Before all experiments, the detector was calibrated (energy calibration, efficiency calibration, and the sample position calibration between the detector and the point source). Within a certain time, the peaks related to the incoming energy photons emitted from the source are formed. The area under these peaks can be calculated using Genie-2000 software, and the rate of this area (calculated area per measuring time) represents the intensity of the incoming photon. The intensity in the absence of the ER sample is represented by the initial intensity ($\:{I}_{0}$), while by placing the ER sample between the detector and the point source, the calculated intensity is represented by the transmitted intensity ($\:I$). From these values, the linear attenuation coefficient (LAC) for a sample of a thickness ($\:x$) can be calculated by the following Eqs^24,25^:

1 $$\:LAC=\frac{1}{x}\:ln\frac{{I}_{0}}{I}$$

The other essential attenuator factors, such as half value length ($\:HVL$), tenth value length ($\:TVL$), Mean free path (*MFP*) and radiation protection efficiency ($\:RPE$) can be expressed by the following equations:

2 $$\:HVL=\frac{Ln\:2}{LAC}$$

3 $$\:TVL=\frac{Ln\:10}{LAC}$$

4 $$\:MFP=\frac{1}{LAC}$$

5 $$\:RPE\%=(1-\frac{I}{{I}_{0}})\times\:100$$

## Results and discussion

**Fig. 3:**
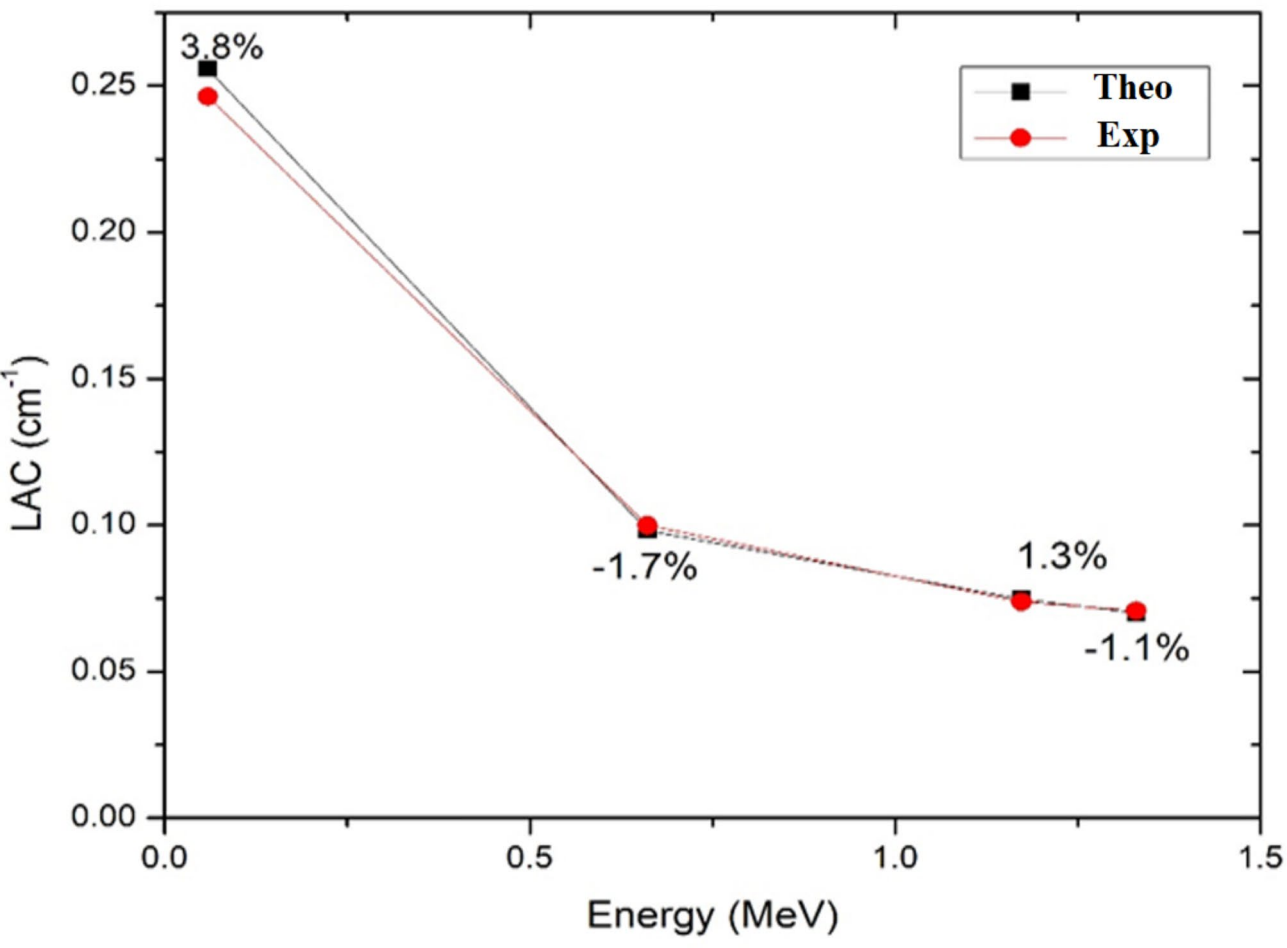
The experimental and theoretical $\:LACs$ of the pure ER sample and the relative deviation.

To assess the radiation shielding efficiency of the designed epoxy resin (ER) samples doped with different concentrations of tin oxide micro and nanoparticles, the experimental method should be validated by estimating the accuracy of the experimental results that can be performed by comparing the pure epoxy resin theoretical values of LAC from PHY-X^26^ by the experimental results. The theoretical and experimental results and their relative deviation are presented in Table 2 and showed in Fig. 3. It is obvious that the PHY-X and experimental outcomes are matched and in good agreement and the relative deviations are sufficiently small ranging from $\:-1.1\:\%$ to $\:3.8\:\%$.

**Table 2:** The experimental (exp) and theoretical (Theo) results of microcomposite (EP-SnO0).

Energy	EP-SnO0		
	Theo	Exp	Dev
0.0595	0.2556	0.2463	3.79
0.6617	0.0982	0.0999	-1.71
1.1730	0.0748	0.0738	1.32
1.3330	0.0700	0.0708	-1.09

The LACs are improved by increasing the concentration of SnO (micro or nano-sized or both sizes) because SnO have higher density than the matrix (Epoxy resin), so it can enhance the probability of the Photoelectric interaction of the incident gamma rays with the samples, on the contrary, the LACs are decreasing by increasing energy for all SnO concentrations due to the Compton scattering and pair production interaction dominate at medium ($\:0.661\:MeV$) and high energy ($\:1.173$ and $\:1.33$) respectively.

The SnO nanoparticles have better LACs than microparticles at all tested energy points. It can be explained as that the agglomerates disintegrate into mixtures containing fine particles, in addition to nanoparticles filling the gaps to provide a higher attenuation efficiency, On the other hand, the density of the epoxy composite from Table 1 in the mixture (micro and nano together) is relatively greater than that of nano or micro as fillers separately, and this gives an indication that the percentage of voids in the $\:0.5$M$\:+0.5$N composite is less. It is worth noting that the effect of the particle size gradually vanishes as the energy increases because the probability that the radiation will penetrate or escape through the sample becomes more significant, so the effect of nanoparticles diminishes gradually. A similar observation was revealed by Cheewasukhanonta et al., who examined the impact of nano and micro bismuth oxide on the attenuation factors for certain glass systems^27^ in addition to the results of Almutairi et al., who investigated the impact of cerium oxide on the attenuation coefficient of silicon rubber which are consistent to the present results^28^.

**Fig. 4:**
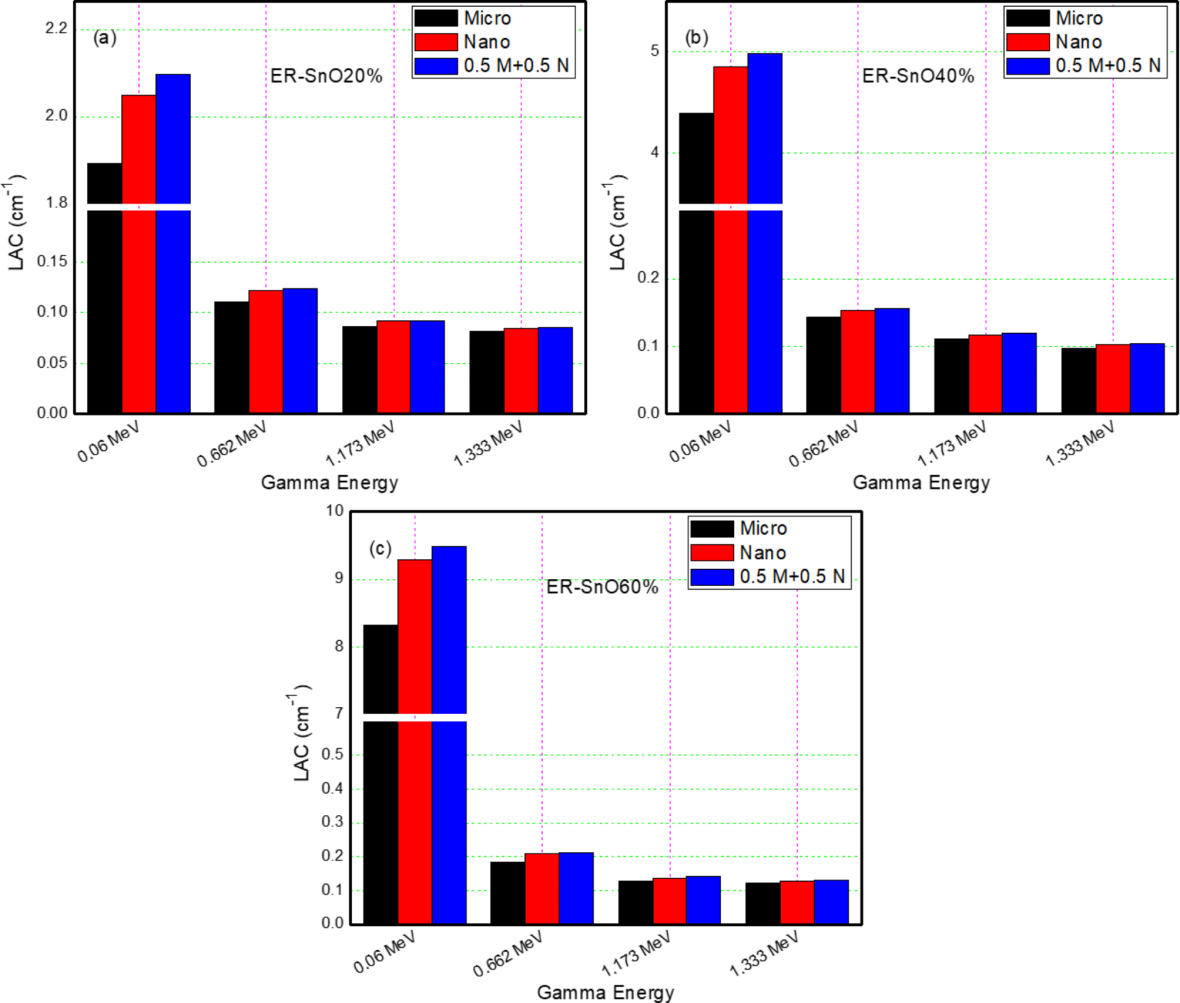
$\:LACs$ of the designed epoxy samples at different gamma energies, (**a**) ER-SnO20%, (**b**) ER-SnO40% and (c) ER-SnO60%.

Considering the ER samples with a SnO combination of micro and nanoparticles, it was observed that the LACs for those samples of weight ratio $\:0.5$ Micro$\::0.5$ Nano is higher than the LACs for the samples with nano SnO or micro SnO each alone and this is at all energy points as shown in Table 3. For example, at energy $\:0.059\:MeV$ The sample of $\:30\%$ micro + $\:30\%$ nano SnO particles (ER-SnO$\:60\%,0.5$M$\:+0.5$N) has exploited the maximum LAC of $\:9.48\:{cm}^{-1}$ defeating the $\:60\%\:$nano SnO sample (LAC$\:=\:9.28\:{cm}^{-1}$) and $\:60\%\:$micro SnO sample (LAC$\:=8.31\:{cm}^{-1})$. Figure 4 illustrates all the above-mentioned outcomes. The cause behind advantage of incorporating both nano and microparticles is the synergistic effect of hybrid nano and microparticles through a material matrix, this effect can be attributed to the dispersing of different particle sizes through the material matrix filling the different sizes voids leading to more uniform and homogenous distribution of SnO particles inside ER samples and increasing chance of radiation interaction.

**Table 3:** The LAC of Epoxy resin composites with different SnO sizes and their relative deviations.

Energy	Micro	Nano	Dev1	0.5 M + 0.5 *N*	Dev2
Exp (EP-SnO20)					
0.0595	1.8936	2.0487	8.19	2.0982	10.80
0.6617	0.1109	0.1212	9.29	0.1232	11.09
1.1730	0.0859	0.0912	6.20	0.0919	6.98
1.3330	0.0815	0.0838	2.82	0.0851	4.42
Exp (EP-SnO40)					
0.0595	4.3884	4.8454	10.41	4.9765	13.40
0.6617	0.1431	0.1529	6.85	0.1561	9.08
1.1730	0.1107	0.1171	5.78	0.1195	7.95
1.3330	0.0979	0.102	4.19	0.1039	6.13
Exp (EP-SnO60)					
0.0595	8.3193	9.2874	11.64	9.4822	13.98
0.6617	0.1839	0.2087	13.49	0.2112	14.85
1.1730	0.1287	0.1367	6.22	0.1411	9.63
1.3330	0.1219	0.1297	6.40	0.1319	8.20

The half value layers (HVLs) and tenth value layers (TVLs) are derivate parameters from LACs. They are inversely proportional to LACs. Less HVL means a better-shielding property. The HVLs and TVLs are increasing by raising the energy and decreasing by increasing the SnO content. Nano SnO samples have fewer HVLs and TVLs than micro samples at all energy and the same SnO concentration. The synergistic effect of nano and micro combinations of SnO is emphasized again. The HVLs and TVLs of the hybrid samples with both nano and microparticles are the highest for all energy values and fixing the SnO content. Table 4 show the relation between the HVL and TVL of ER samples embedded by different concentrations and particle size and gamma energy respectively.

**Table 4:** The HVL and TVL of different prepared epoxy resin composites.

	Energy	EP-SnO0	Exp (EP-SnO20)			Exp (EP-SnOO40)			Exp (EP-SnO60)		
			Micro	Nano	0.5 M + 0.5 N	Micro	Nano	0.5 M + 0.5 N	Micro	Nano	0.5 M + 0.5 N
HVL, cm	0.0595	2.7114	0.3660	0.3383	0.3304	0.1579	0.1431	0.1393	0.0833	0.0746	0.0731
	0.6617	7.0588	6.2502	5.7190	5.6262	4.8438	4.5333	4.4404	3.7692	3.3213	3.2819
	1.1730	9.2700	8.0692	7.5978	7.5424	6.2615	5.9193	5.8004	5.3858	5.0706	4.9125
	1.3330	9.8983	8.5049	8.2714	8.1451	7.0802	6.7956	6.6713	5.6862	5.3442	5.2551
TVL, cm	0.0595	9.0070	1.2160	1.1239	1.0974	0.5247	0.4752	0.4627	0.2768	0.2479	0.2428
	0.6617	23.4487	20.7627	18.9982	18.6898	16.0907	15.0594	14.7507	12.5209	11.0330	10.9024
	1.1730	30.7942	26.8054	25.2393	25.0553	20.8002	19.6634	19.2685	17.8911	16.8441	16.3188
	1.3330	32.8814	28.2526	27.4771	27.0574	23.5198	22.5744	22.1616	18.8891	17.7532	17.4571

Considering the other shielding parameters such as half value layer (HVL) and tenth value layer (TVL) of the composites under study, they were decreased with increasing tin oxide concentration in the form of nano, micro, or combined sizes of particles. Also, the composites prepared using both sizes of SnO (nano and micro) were found to have the least HVL and TVL followed by the samples prepared using nanoparticles of SnO, and finally, the samples prepared using microparticles of SnO which had the highest HVL and TVL values. It is worth mentioning that the proposed epoxy/SnO composites with $\:30\text{\%}$ content of SnO micro, nano, or a combination of both sizes of particles offer approximately $\:100\text{\%}$ radiation protection efficiency (RPE) at low energy $\:0.059\:MeV$. Overall, epoxy/tin oxide composites offer an efficient novel shielding material against ionizing radiation, this is particularly valuable and suitable for so-called low-energy applications such as medical imaging and radiation therapy.

The mean free path (MFP) represents the average free distance of a photon inside the material before any collisions with atoms. The greater this distance, the more it indicates the weakness of the material as an attenuator or absorber against photons and vice versa. The lowest MFP in the discussed epoxy composites at $\:0.662\:MeV$ was $\:4.7348\:cm$ for EP-SnO$\:60$ ($\:0.5$M$\:+0.5$N) while the largest MFP at the same energy was $\:10.1837$ for EP-SnO0. The values of MFP results at other discussed energies were tabulated in Table 5.

**Table 5:** The mean free path of prepared composites from 0.060 to 1.333 MeV.

	Energy	EP-SnO0	Exp (EP-SnO20)			Exp (EP-SnOO40)			Exp (EP-SnO60)		
			Micro	Nano	0.5 M + 0.5 N	Micro	Nano	0.5 M + 0.5 N	Micro	Nano	0.5 M + 0.5 N
MFP, cm	0.0595	3.9117	0.5281	0.4881	0.4766	0.2279	0.2064	0.2009	0.1202	0.1077	0.1055
	0.6617	10.1837	9.0171	8.2508	8.1169	6.9881	6.5402	6.4061	5.4377	4.7916	4.7348
	1.1730	13.3737	11.6414	10.9613	10.8814	9.0334	8.5397	8.3682	7.7700	7.3153	7.0872
	1.3330	14.2802	12.2699	11.9332	11.7509	10.2145	9.8039	9.6246	8.2034	7.7101	7.5815

**Fig. 5:**
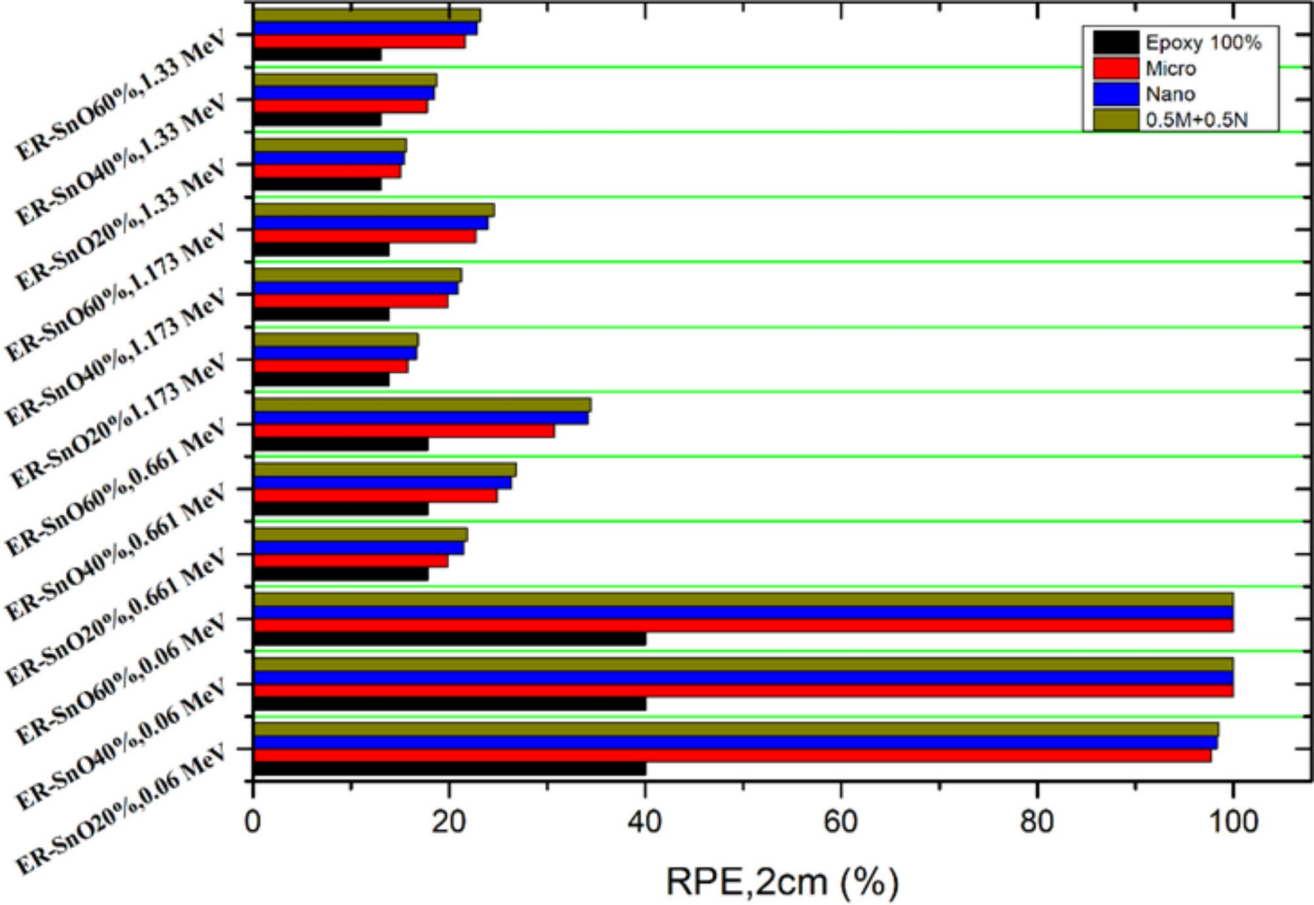
The $\:RPE$ of ER with micro and nano SnO and the ER with SnO in both sizes at various energies.

The last important factor is radiation protection efficiency (RPE) at the practical operational thickness of $\:2\:cm$, it provides us with complete figures for the radiation protection capability of the produced samples. From Fig. 5 it can be concluded that the maximum efficiencies of ER-SnO samples are at $\:0.059\:MeV$ with approximately $\:100\%$ radiation absorption efficiency, then the efficiency decreased to about $\:34.45\:\%$ for ER-SnO samples doped by $\:30\%$ microparticles and $\:30\%$ nanoparticles of SnO at radiation energy of $\:0.661\:MeV$. Again, the most efficient samples are hybrid samples incorporating $\:30\%$ microparticles and $\:30\%$ nanoparticles of SnO with $\:24.59\%$ and $\:23.19\%$ for $\:1.173\:MeV$ and $\:1.33\:MeV\:$respectively. Table 6 display the RSE of different thickness composites at different energies.

**Table 6:** The RSE of different thickness composites (2, 4 and 6 cm) at different energies.

	Energy	EP-SnO0	Exp (EP-SnO20)			Exp (EP-SnOO40)			Exp (EP-SnO60)		
			Micro	Nano	0.5 M + 0.5 N	Micro	Nano	0.5 M + 0.5 N	Micro	Nano	0.5 M + 0.5 N
RSE, % (2 cm)	0.0595	40.03	97.73	98.34	98.50	99.98	99.99	100.00	100.00	100.00	100.00
	0.6617	17.83	19.89	21.53	21.84	24.89	26.35	26.82	30.77	34.12	34.45
	1.1730	13.89	15.79	16.68	16.79	19.86	20.88	21.26	22.69	23.92	24.59
	1.3330	13.07	15.04	15.43	15.65	17.78	18.45	18.76	21.64	22.85	23.19
RSE, % (4 cm)	0.0595	64.03	99.95	99.97	99.98	100.00	100.00	100.00	100.00	100.00	100.00
	0.6617	32.48	35.83	38.42	38.91	43.58	45.75	46.44	52.08	56.60	57.04
	1.1730	25.85	29.08	30.57	30.76	35.78	37.40	38.00	40.24	42.12	43.13
	1.3330	24.43	27.82	28.48	28.85	32.40	33.50	34.01	38.59	40.48	41.00
RSE, % (6 cm)	0.0595	78.43	100.00	100.00	100.00	100.00	100.00	100.00	100.00	100.00	100.00
	0.6617	44.52	48.59	51.67	52.25	57.62	60.04	60.80	66.83	71.41	71.84
	1.1730	36.15	40.27	42.15	42.39	48.53	50.47	51.18	53.80	55.97	57.11
	1.3330	34.31	38.68	39.52	39.99	44.42	45.77	46.39	51.88	54.08	54.68

**Fig. 6:**
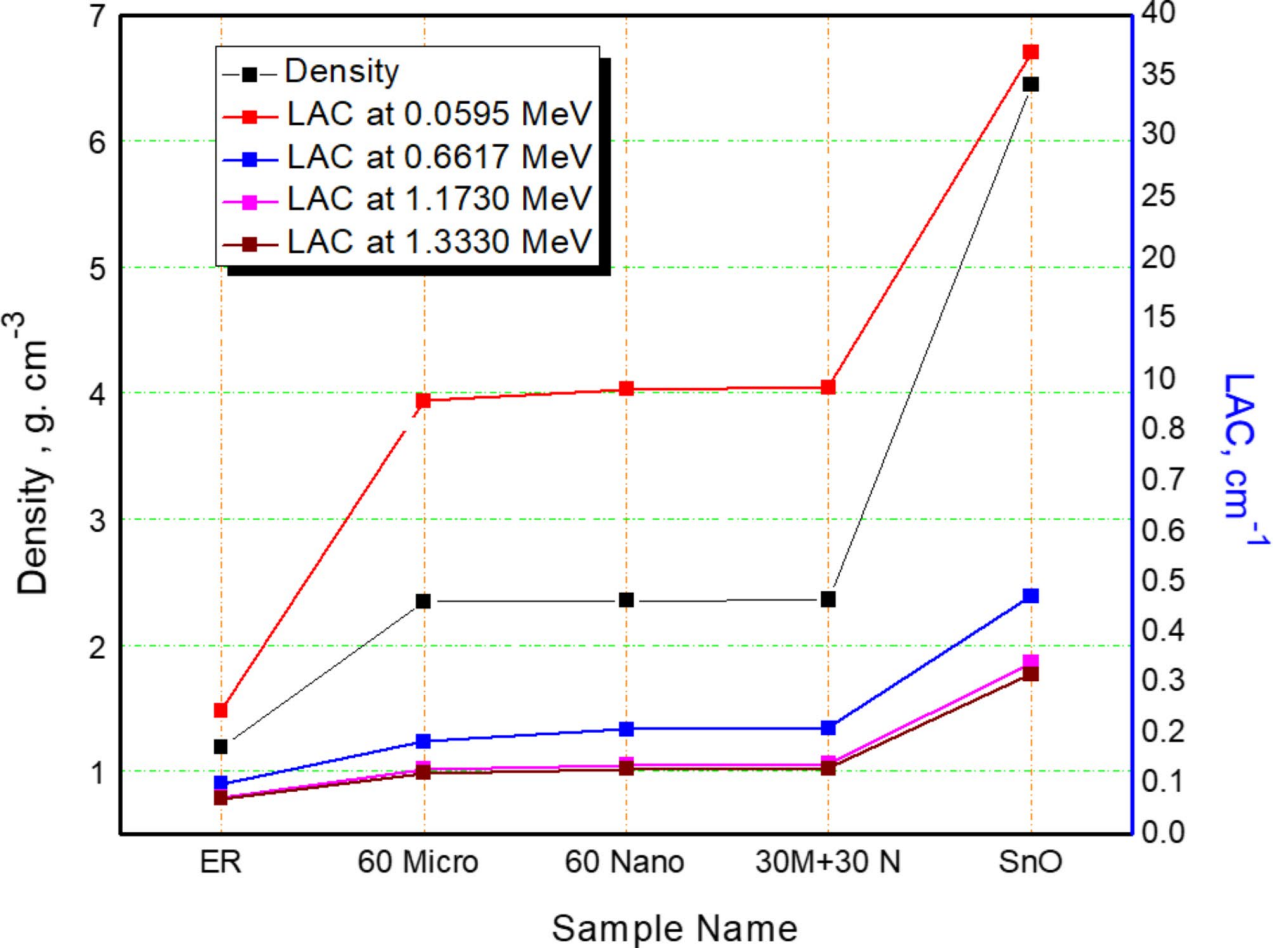
The variation of density and LAC with Pure Epoxy, pure SnO and SnO/Epoxy resin composites.

The density and linear attenuation coefficient (LAC) for the effective present composites (EP-SnO $$\:60\%$$ (Micro, Nano and $$\:0.5$$M$$\:+0.5$$N)) and compared with lower bound (Pure epoxy resin) and upper bound (SnO) as shown in Fig. 6 The deviation is clear at low energy between the upper and lower limits, so we found that the values of the LAC were $$\:0.2463,\:8.3193,\:9.2874,\:9.4822\:$$and$$\:\:36.933$$$$\:{cm}^{-1}$$ due to the variation of density from $$\:1.25$$ to $$\:6.45$$$$\:g.{cm}^{-3}\:$$as well as the k-edge of the Sn-element at $$\:37$$$$\:keV$$. At medium and high energies, the deviation is not large between the prepared samples and the lower and upper bound.

The lead and bismuth equivalent thickness of the present composites of thickness 2 cm was calculated according to next formula:

6 $$\:\left({d}_{Pb}\right)=\frac{{LAC}_{ER-SnO}}{{LAC}_{Pb}\:}\:*\:{d}_{ER-SnO}\:\:$$

The results of heavy metal equivalent thickness were calculated for the highest attenuator composites contaning both micro and nano fillers and reported in Table 7. The results showed, for example, at high energy (1.333 MeV), that the thickness of ER-SnO60% required to attenuate about 93% of this energy is 4 times the thickness required for lead to attenuate the same percentage. Note that lead is a toxic material that is not environmentally friendly, in addition to the high cost of forming a lead wall, this indicates the importance of the current work, as samples with a large thickness can be used, but they are environmentally safe and inexpensive, in addition to the aesthetic appearance of the samples compared to lead and bismuth in places that use such energies, such as radiotherapy centers, nuclear reactors and accelerators.

**Table 7:** The lead and bismuth equivalent thickness at different gamma-ray energies.

Lead and Bithmus Equivalent Thickness	Eneergy (MeV)	ER-SnO20%		ER-SnO40%		ER-SnO60%	
		0.5 M + 0.5 N		0.5 M + 0.5 N		0.5 M + 0.5 N	
		d_Pb_ (mm)	d_Bi_ (mm)	d_Pb_ (mm)	d_Bi_ (mm)	d_Pb_ (mm)	d_Bi_ (mm)
	0.0595	0.80	0.89	1.89	2.10	3.60	4.01
	0.6617	2.10	2.38	2.66	3.02	3.59	4.09
	1.173	2.72	3.11	3.53	4.05	4.17	4.78
	1.333	2.75	3.16	3.36	3.86	4.27	4.90

## Conclusion

The present study was performed to estimate the radiation shielding characteristics of fabricated epoxy resin (ER) composites embedded with tin oxide (SnO) in different concentrations and particle sizes. The experimental approach was employed to find the linear attenuation coefficient (LAC) for each of the prepared ER samples and then calculate the other shielding parameters using the determined values of LAC. Increasing SnO content led to enhancing the shielding performance of the epoxy/tin oxide composites in terms of LAC. The proportionality relation between LACs and the concentration of SnO nano and/or microparticles in the composite was highly distinguished in the low-energy radiation zone, on the other hand, it wasn’t as noticeable in the high-energy zone. Beyond that, the results revealed that the fabricated composites which contain both micro and nano sizes of SnO particles showed a superior performance in shielding. The LACs for epoxy/tin oxide composites that have been prepared with SnO combination of micro and nanoparticles (weight ratio of $\:0.5\:$Micro:$\:0.5$ Nano) is higher than the LACs of the samples with nano or micro SnO particles each alone and this is in all energy ranges. This finding recommends using the lower-cost epoxy/tin oxide composites made of both micro and nano-sized particles rather than using the composites containing only nano-sized particles.

## Data Availability

The datasets used and/or analysed during the current study available from the corresponding author on reasonable request.
